# Organic–Inorganic Metal Halide Perovskites: Toward Stability, Chirality, and AI-Guided Discovery

**DOI:** 10.1021/acscentsci.5c02169

**Published:** 2026-01-26

**Authors:** Jiaonan Sun, Yiying Wu

**Affiliations:** † School of Chemical Engineering and Light Industry, 47870 Guangdong University of Technology, Guangzhou 510006, China; ‡ Department of Chemistry and Biochemistry, 2647 The Ohio State University, Columbus, Ohio 43210, United States

## Abstract

Organic–inorganic metal halide perovskites (OIMHPs) are rapidly emerging as a versatile class of hybrid semiconductors, driven in part by the development of next-generation solar cells based on metal halide perovskites. By integrating the structural flexibility of organic components with the inorganic lattices, OIMHPs offer unprecedented opportunities for designing materials with tailored optoelectronic functionality, stability, photophysics, and spintronic properties. In this outlook, we highlight three frontier directions shaping the future of low-dimensional OIMHPs: (i) incorporation of conjugated cations to tune charge transport, energy alignment, and stability, (ii) introduction of chirality to enable chiral-induced spin selectivity and spintronic devices, and (iii) application of artificial intelligence to accelerate structure prediction and discovery. Lastly, we also outline critical challenges, including the need for standardized stability benchmarks, spin functionality, and robust data sets for machine learning. Addressing these challenges will not only advance OIMHP chemistry but also unlock transformative applications spanning photovoltaics, spintronics, and photoelectrocatalysis.

## Graphical abstract

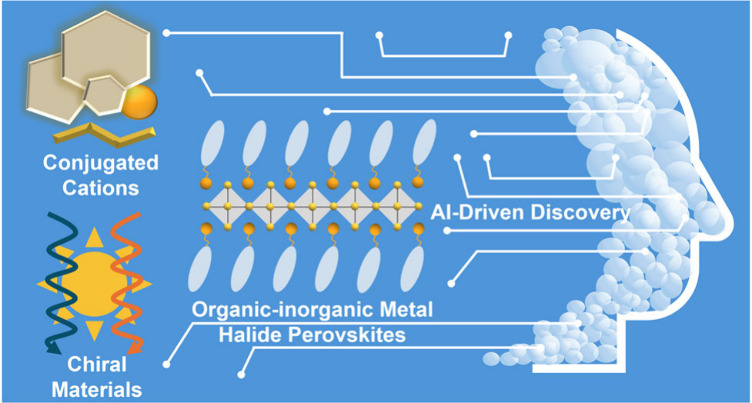

## Introduction

Organic–inorganic metal halide perovskites (OIMHPs) constitute a highly versatile class of hybrid semiconductors in which flexible, functional organic cations are integrated with ordered inorganic lattices.^1–3^ This structural duality enables unprecedented tunability over optoelectronic behavior, photophysics, stability, and spin properties. As a result, OIMHPs have rapidly emerged as a broad materials platform whose application potential extends far beyond their perovskite origins—including photovoltaics,^4^ light-emitting diodes,^5,6^ field-effect transistors,^7^ spin-selective systems,^8,9^ and quantum materials.^10^

The rapid expansion of the field of OIMHP research has been closely intertwined with the meteoric rise of halide perovskites in photovoltaics. In the ABX_3_ structure—where A is a monovalent organic cation, B a divalent metal, and X a halide—hybrid perovskites proved to be outstanding light absorbers, catalyzing a paradigm shift in next-generation solar cells.^11–13^ Pioneering work by Miyasaka and co-workers^14^ first demonstrated halide perovskites as sensitizers in dye-sensitized solar cells. Later, 2,2′,7,7′-tetrakis­(*N*,*N*-di-*p*-methoxyphenylamine)-9,9′-spirobifluorene (spiro-OMeTAD) was adopted in an all-solid-state solar cell configuration by Park et al., achieving a power conversion efficiency (PCE) of 9.7%.^15,16^ Within a few decades, the PCE of perovskite solar cells (PSCs) has been propelled to near 27%,^17^ accompanied by the surge of perovskite-related industry efforts to actively scale up cell size and develop single-junction cells, tandem structures, flexible devices, and more.

Despite the impressive performance of three-dimensional (3D) lead halide perovskites, several persistent challenges remain. Derived from the concept of “liquid crystals”, 3D perovskites exhibit a unique “crystal liquid” duality, characterized by a soft lattice and dynamic ionic structure.^18^ This leads to phenomena such as defect generation, ion migration, phase degradation, and pronounced sensitivity to external stimuli—including light, heat, moisture, pressure, and electrical bias. As a result, perovskite devices often suffer from instability and a limited operational lifespan, posing significant barriers to their long-term commercial deployment.^19^

OIMHPs provide a new material paradigm for solving the inherent instability by extending the backbone beyond conventional 3D perovskites, contributing significantly to the long-term stability and imparting novel functionalities of perovskite-based applications. Two-dimensional (2D) perovskites are formed when inorganic metal halide octahedral sheets are sandwiched between organic cations. The bulky organic moieties within the low-dimensional structure can serve as barriers for ion migration, moisture, oxygen, etc. One-dimensional (1D) perovskites consist of metal halide octahedra connected through face-, edge-, and corner-sharing, resulting in chain structures. Isolated metal halide octahedra, separated by organic cations, constitute zero-dimensional (0D) perovskites. Due to the relaxation of the Goldschmidt tolerance factor, perovskite can accommodate different cations with either conjugated backbone,^20^ chromophore,^21^ chiral molecules,^22^ polymerizable monomers,^23^ or aggregation-induced emission molecules.^24^ Moreover, exotic all-organic 2D perovskite and high-entropy halide perovskite single crystals can be prepared.^25,26^ Reversely, perovskite can be exfoliated into monolayers to form Moiré patterns through stacking^27^ or confined into nanostructures like carbon nanotubes, which induce the formation of a chain structure with physical barriers.^28,29^

OIMHPs can accommodate a wide range of exotic organic cations, including conjugated moieties.

Given their structural diversity and versatility, we tend to consider the use of the OIMHPs as a platform for tailored materials, not just solar absorbers. There is a pressing need for new material paradigms beyond conventional 3D lead halide perovskites. Equally important is advancing our understanding of design rules to predictively synthesize new structures from organic cations, considering the synergies among conjugated systems, chiral components, and artificial intelligence (AI)-assisted approaches.

In this Outlook, we highlight three frontier directions that illustrate the transformative potential of OIMHPs (Figure 1). (i) Incorporation of conjugated cations to tune charge transport, energy alignment, and stability. Conjugated cations can expand the OIMHP family, enabling new materials that bridge the stability gap for photovoltaics while remaining compatible with 3D perovskite lattices. (ii) Introduction of chirality to enable chiral-induced spin selectivity and spintronic devices. Direct incorporation of chiral organic molecules imparts homochirality to the inorganic lattice and offers unprecedented tunability. (iii) Application of AI to accelerate structure prediction and discovery. The use of AI tools can significantly accelerate the discovery of OIMHP, particularly given the vast combinatorial space of organic and inorganic components. Finally, we discuss the key remaining challenges and outline prospective directions for future research. Together, these approaches point toward a new generation of hybrid semiconducting materials with expanded capabilities across energy, spintronics, and catalysis.

**Figure 1:**
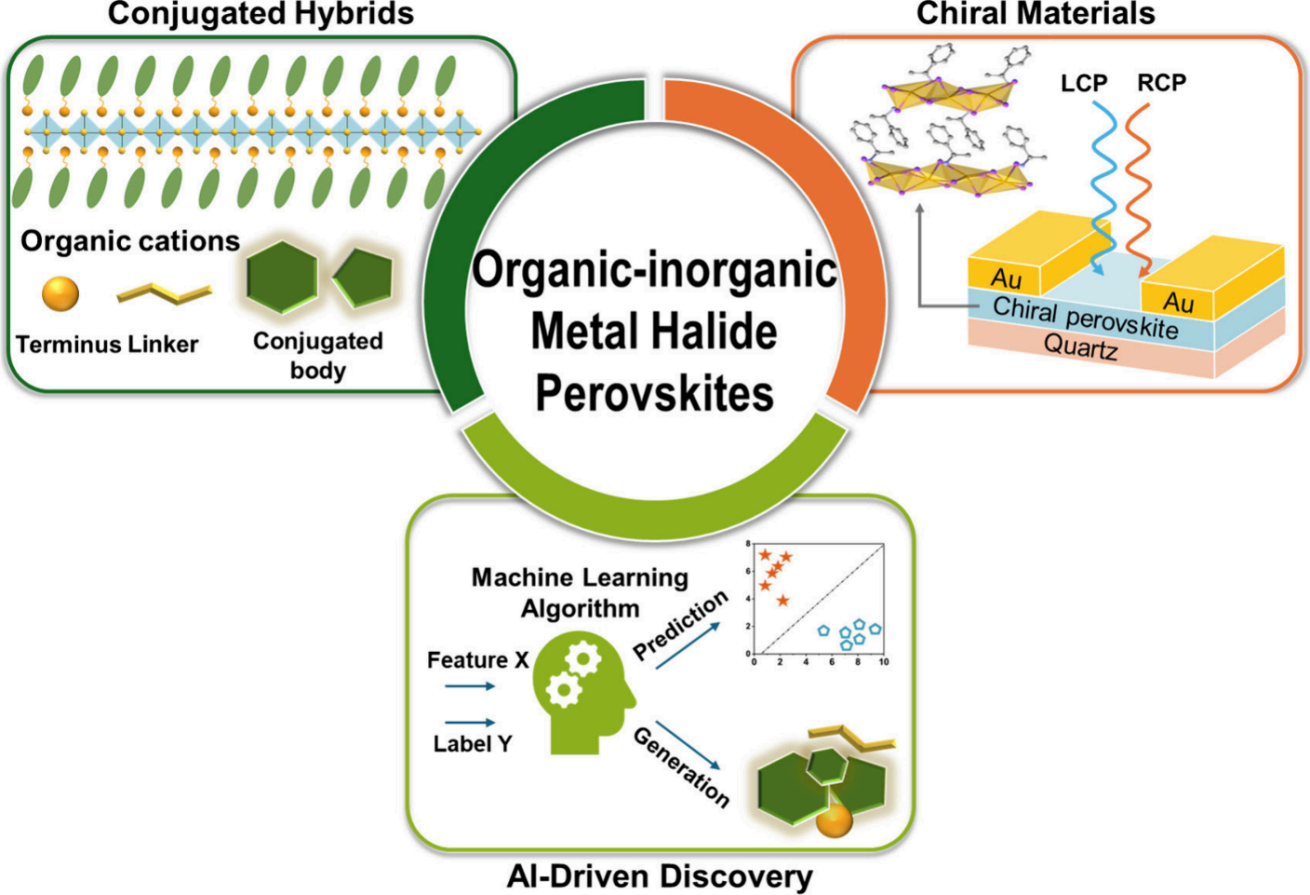
Organic–inorganic metal halide perovskites with synergies among conjugated hybrids, chiral materials, and AI-driven discovery.

## Low-Dimensional Conjugated Hybrids

The inclusion of conjugated organic cations brings exciting changes in optoelectronic properties, energy landscape, ionic properties and stability. Here, we specifically focus on energy landscape management and stability.

OIMHPs typically adopt molecular quantum-well structures due to the quantum and dielectric confinement, where organic molecules serve as barriers, and the inorganic lattice functions as wells. Organic molecules, with varied degrees of conjugation and frontier orbitals, can contribute to the band structures of the hybrids, leading to diverse energy level alignments. As shown in Figure 2a, in a type-I heterostructure, the organic spacer has a HOMO–LUMO gap wider than the gap between the valence band (VB) and conduction band (CB) edges of the inorganic framework. OIMHPs with type-I band alignment exhibit strong photoluminescence due to the exciton recombination confined within the inorganic lattice. By increasing conjugation of the organic cations, the HOMO or LUMO levels can be tuned to form staggered type-II heterostructures. In such cases, charge carriers can be extracted from the inorganic framework to the organic moieties, resulting in photoluminescence quenching. In reverse type-I heterostructures, the HOMO–LUMO gap lies between the VB and CB energy levels, and energy transfer occurs from the inorganic lattice to the organic layer, resulting in emission entirely from the organic moiety. Typical examples for reverse type-I energy alignment include BTm^30^ and AE4T^31^ cations. Among them, using a molecular engineering approach to build type-II 2D/3D heterostructures can be applied to solar cells, enabling improved interfacial charge extraction and carrier transport.

**Figure 2:**
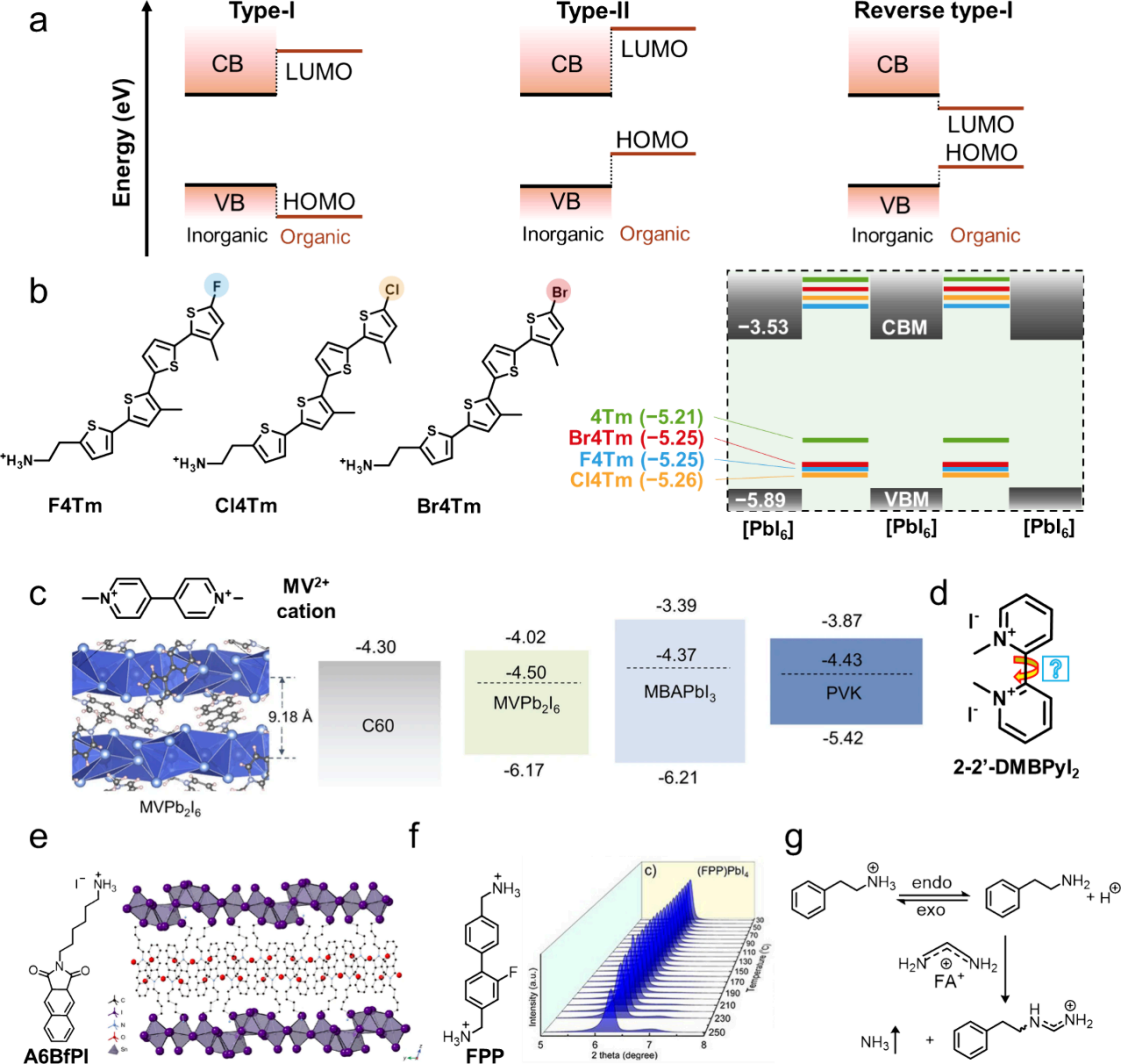
(a) Energy scheme of type-I, type-II, and reverse type-I alignments. (b) Molecular design of quaterthiophene-based cations designed for energy level tuning. Reproduced from ref 35. Available under a CC-BY 4.0 license. Copyright 2023 The American Association for the Advancement of Science Ma et al. (c) MV^2+^ cation and the resulting 1D MVPb_2_I_6_ for energy level tuning in inverted perovskite solar cells. Reproduced from ref 32. Available under a CC-BY 4.0 license. Copyright 2024 Springer Nature Li et al. (d) Organic cations exhibiting varied dihedral angles. Reproduced from ref 36. Available under a CC-BY 4.0 license. Copyright 2024 Wiley-VCH GmbH Patra et al. (e) The A6BfPI molecule and its 2D perovskitoids with a new octahedral connectivity. Reproduced with permission from ref 43. Copyright 2024 Springer Nature. (f) FPP cation and its 2D perovskites with enhanced thermal stability, as evidenced by variable-temperature X-ray diffraction patterns. Reproduced from ref 44. Copyright 2021 American Chemical Society. (g) Reaction scheme between phenethylammonium and formamidinium cations. Reproduced with permission from ref 47. Copyright 2023 Springer Nature.

Representative cations for type-II heterostructures include thiophenes,^30^ viologens,^32^ and pyrene-based organic cations.^33^ Dou and co-workers designed a series of quaterthiophene-based organic cations (Figure 2b) with shallow HOMO for interfacial hole extraction between the perovskite active layer and hole-transporting layer (HTL).^34,35^ Adding an electron-withdrawing group further realized a dedicated control of interfacial energy alignment, minimized the energy barrier for hole extraction, and constructed highly efficient and stable PTAA-based n-i-p perovskite devices. Manipulating the penetration depth through π-conjugated pyrene-based organic cations also contributes to the surface band-edge structures, increasing hole mobilities and device PCE.^33^ In inverted architecture, the use of methylviologen (MV^2+^) cations with deep LUMO also forms type-II heterostructures, facilitating the electron transport between C60 and 3D perovskite.^32^ Tuning the n value (the number of perovskite layers) through twisted molecular conjugation with varied dihedral angles could lead to various energy alignments for potential device applications (Figure 2d).^36,37^ Other than the conjugated bodies, the terminus group and linkers, having the potential of forming hydrogen bonds or affecting the penetration depths, also offer the opportunities of modulating the band structures of hybrids.^38,39^

With organic intercalating cations, low-dimensional OIMHPs are typically more stable than 3D structures. This enhanced stability arises from the organic moieties encapsulating the inorganic framework, effectively acting as protective layers. Nevertheless, stability varies significantly in regard to different organic cations. For example, in the 2D (4Te)_2_PbI_4_ perovskite, the as formed low-dimensional OIMHPs can remain stable for months when immersed in water.^40^ However, some OIMHPs containing conjugated cations, such as PEA^+^, have limited stability.^41^ The design of the quaterthiophene molecules with ethyl side chains is key to enhancing the hydrophobicity, thereby significantly improving the aqueous stability. The conjugated backbone and aliphatic side-chains jointly enhance the material’s stability. 1D perovskite, MVPb_2_I_6_, has even been employed as light absorbers in photocatalytic systems.^42^ 2D perovskitoids with unique octahedral connectivity (including corner-, edge- and face-sharing) within the inorganic sheets, driven by the design of organic cations, also improved the device operational stability (Figure 2e).^43^ This stability enhancement mainly arises from the changes in inorganic lattice connectivity, especially the introduction of face-sharing octahedra, which tend to be more stable than corner-sharing-only structures. Thermally, the temperature threshold for decomposition is also higher for OIMHPs, can be up to 200–300 °C, as determined by techniques such as thermogravimetric analysis^41^ or temperature-dependent X-ray diffraction (Figure 2f).^44^ This thermal stability is influenced by the π-π-stacking interactions of the conjugated organic units. Upon heating, halide interdiffusion visualized by emission is also significantly slower in the heterostructures of OIMHPs compared with hybrids containing aliphatic cations.^45,46^ The design of ammonium or amidinium termini, characterized by different p*K*_a_, also garnered much attention due to the improved thermal stability by retarding the deprotonation process (Figure 2g).^47–50^ These examples demonstrated that the stability of OIMHPs is associated with both the organic units and the inorganic sublattices. In our view, enhancing the conjugation of organic moieties, introducing hydrophobic side-chains, tuning interlayer secondary interactions, and increasing the p*K*_a_ of the cation terminus represent decisive and broadly applicable strategies for achieving robust stability of organic–inorganic perovskite hybrids.

From the perspective of binding strength, there is still some debate over the stability of RP- and DJ-phase 2D perovskites along with cation diffusion problems. The major difference is that the DJ-phase has divalent cations, while the RP-phase has monovalent cations. It has been reported that organic cations with multiple strong bonding site with the addition of an electron-withdrawing group can enhance the anchoring and prevent cation migration.^44,51^ On the other hand, RP-phase 2D perovskite, having Van der Waal gaps between cation layers, with only one binding site but less lattice distortion, provides more hydrophobicity and enhances environmental stability.^52,53^ Due to the limited commercial cation pools, further synthetic efforts can be applied to systematically compare the material stability, tailored in *2D*/3D heterostructure configurations and devices.

## Chiral OIMHPs

Chiral perovskite materials have attracted growing interest due to their circular dichroism, circularly polarized light (CPL) emission,^54^ ferroelectricity and spintronic properties.^55–57^ They have important applications such as CPL photodetectors and chiral spintronics.^8,58–60^ Using chiral cations or creating a chiral environment through assembly or a chiral agent is the most common strategy to obtain chiral perovskites. When using chiral organic cations for OIMHPs, the enantiomeric purity is defined by the chiral organic cation type, while the electronic properties are defined by the inorganic components. Due to the soft ionic nature, the hybrid perovskite framework can easily accommodate chiral cations to allow a tunable chirality in a dynamic functional inorganic scaffold. The first 1D chiral perovskite single crystal is using S-methylbenzylammonium (MBA) as organic cation with a formula of ((S)-MBA)­PbBr_3_, reported by Billing and Lemmerer.^22^ Controlling the cooling temperature, 2D chiral perovskite single crystals with the same methylbenzylammonium cation can be obtained with a formula of (R-/S-MBA)_2_PbI_4_ (Figure 3a).^61^ Furthermore, more chiral cations, such as 1-(4-chlorophenyl)­ethylammonium, 3-ammoniopyrrolidinium, 3-fluoropyrrolidinium, and 1,2-diaminocyclohexane were used as chiral cations to produce chiral OIMHPs.^55,62^ The related properties of chiral OIMHPs, including chiroptical properties, Rashba splitting, ferroelectricity, and quantum properties were further studied.^55^ The important performance parameters, including anisotropic factors and photoluminescence quantum yield (PLQY), have been summarized in earlier published reviews.^63^

**Figure 3:**
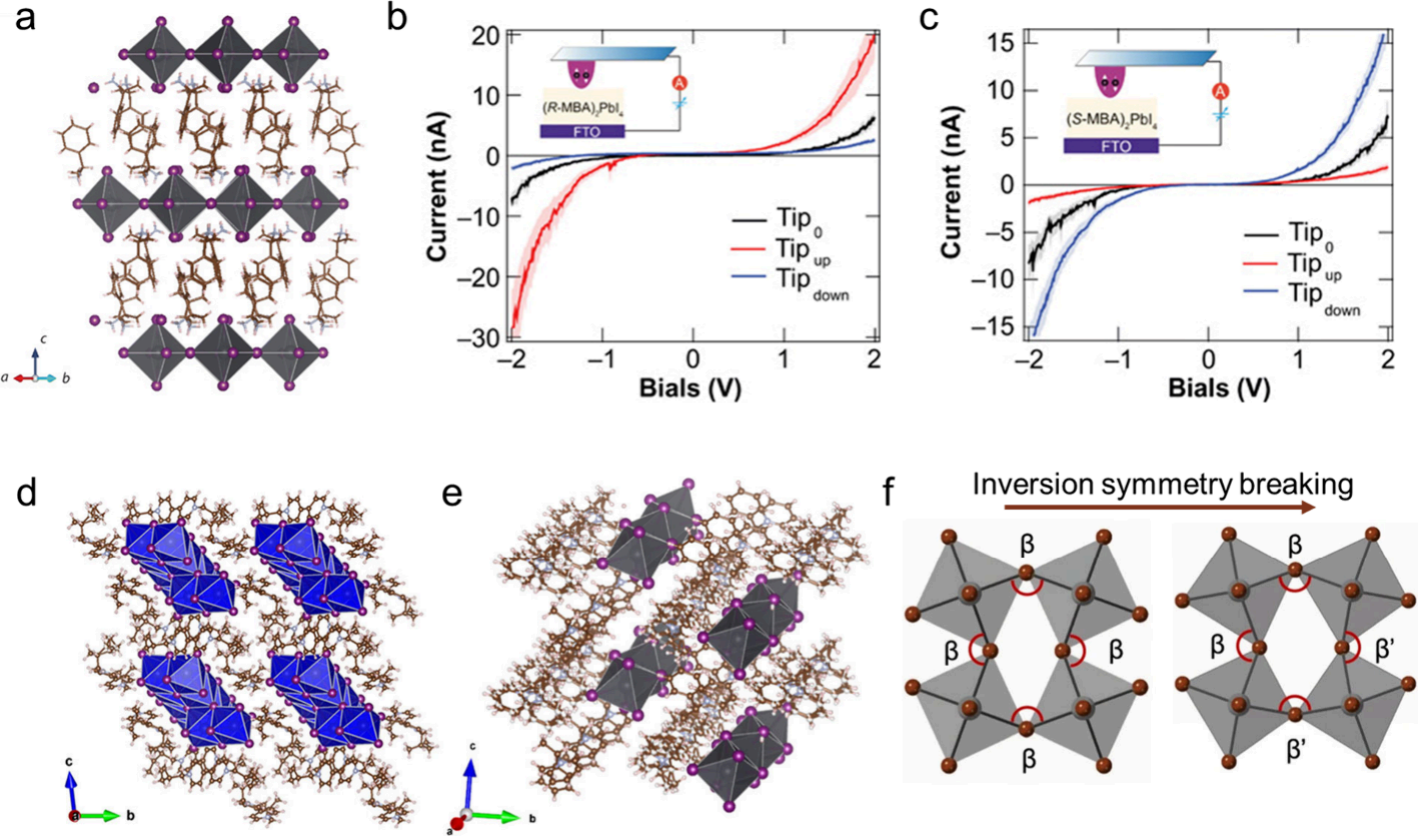
(a) Crystal structure of (R-/S-MBA)_2_PbI_4_. (b) Magnetic conductive-probe AFM measurement on (R-MBA)_2_PbI_4_ perovskite. (c) Magnetic conductive-probe AFM measurement on (S-MBA)_2_PbI_4_ perovskite. Reproduced from ref 9. Available under a CC-BY 4.0 license. Copyright 2019 The American Association for the Advancement of Science Lu et al. (d) Crystal structure of (R-/S-MBV)_2_Pb_5_I_14_. Reproduced from ref 69. Copyright 2024 American Chemical Society. (e) Crystal structure of (R-MBnP)­PbI_3_. Reproduced from ref 70. Copyright 2025 American Chemical Society. (f) Schematics of inversion symmetry breaking of metal-halide octahedrons. Reproduced from ref 72. Available under a CC-BY 4.0 license. Copyright 2021 Springer Nature Jana et al.

### Chiral-Induced Spin Selectivity (CISS)

One of the interesting properties of 2D or 1D OIMHPs is the chiral-induced spin selectivity (CISS) effect, which was introduced by Waldeck and Naaman in 1999,^64^ that contributes to chiral spintronics applications. CISS occurs when electrons travel through chiral materials, where the handedness of chiral molecules dictates the spin of transmitted or emitted electrons. A defining characteristic of the CISS effect is its ability to manipulate electron spin without an external magnetic field and its ability to operate at room temperature. The first demonstration of spin-dependent charge transport in chiral halide perovskite semiconductors was in 2019, by Lu et al.^9^ On parallel-oriented (MBA)_2_PbI_4_ thin films, the measured polarized current shows different responses depending on the magnetization direction of the magnetic conductive-probe atomic force microscope (AFM) tip (Figure 3b and 3c). Kim et al. first demonstrated a spin LED with a chiral halide perovskite, featuring the generation of circularly polarized electroluminescence by CISS.^8^ Other interesting demonstration including the integration of III–V semiconductors with chiral OIMHPs enabling spin control across the interface,^65^ core–shell nanocrystals with achiral core and chiral shell and spin-funneling mechanism for polarized emission.^66^ The evaluation of CISS can be achieved by fabricating these spintronics including spin LED, spin valves, and circularly polarized photodetectors, and concurrently the guidelines for CISS in chiral OIMHPs demand further systematic study.

### Chiral Transfer

In terms of the incorporation of the chiral organic cations, a natural but difficult question is how to design the organic cations to realize efficient chiral transfer to the whole hybrid system and achieve sizable spin-splitting. Methylviologen lead iodide was initially reported as a type-II 1D OIMHP with charge transfer from the inorganic part to the organic part.^67,68^ Wu and co-workers introduce chiral substituents at the N sites, yielding enantiopure viologen derivatives, which were subsequently used to synthesize chiral viologen-based 1D perovskites (Figure 3d).^69^ Through successful chiral crystallization, these materials have a wide circular dichroism response and excellent aqueous stability, demonstrating promising potential for chiral optoelectronics. Another chiral construction strategy is through the Zincke reaction on pyridine (Figure 3e).^70^ The as-formed methylbenzylpyridinium-based chiral OIMHPs demonstrate successful chirality transfer from the pyridinium to the inorganic part, showing excellent photoluminescence and CPL at room temperature.

While several enantiopure organic cations for chiral OIMHPs have been reported, an in-depth study of chirality transfer from the organic to the inorganic component was conducted by Jana et al.^71^ In (naphthyl)­ethylammonium (NEA) based chiral 2D perovskites, the chiral NEA forms hydrogen bonding with the Pb–I octahedra and leads to asymmetric helix structural distortion of the inorganic lattice. The structural asymmetry and spin–orbit coupling together contribute to enhanced Rashba spin splitting. Other than the NEA example, they further summarized that the specific bond angle disparity (β and β′, Figure 3f), which quantifies the structural distortions, is the key descriptor of spin-splitting in 2D OIMHPs.^72^ Both the Rashba effect and the CISS effect are important properties of chiral OIMHPs. Systematically varying the cation structure, tuning the interlayer steric hindrance, as well as developing fast-screening descriptors for enhancing chirality transfer and spin selection using a vast database will be promising research avenues going forward.

## AI-Driven Discovery

Machine learning, as a powerful computational tool, has demonstrated strong predictive capabilities in fields such as materials science, catalyst development, drug molecule design, and theoretical chemistry.^73–80^ Representative large language models, such as ChatGPT, have significantly changed the way in which learning, writing, and teaching are conducted.^81^ A machine learning model is trained to identify the parameters that maximize the likelihood of the observed data. The conventional pathway for designing new materials largely relies on expensive trial-and-error testing. In contrast, the development of AI in materials science, coupled with high-throughput computational screening, enables the rapid establishment of quantitative structure–property relationships by analyzing vast material databases. It can not only predict outcomes but also optimize formulations and pathways. This approach reduces the cost, minimizes manpower requirements, and significantly improves the efficiency, thereby providing important guidance for the development of new materials.

The development and discovery of OIMHPs can be accelerated through machine learning by deepening our understanding of both their physical and their chemical properties. Although significant progress has been made, current machine-learning-driven applications in the field of perovskites are still scratching the surface with many unresolved problems and theoretical challenges. With the rapid growth of perovskite research publications, the most straightforward approach is to use the existing literature as a data set for model training. Based on reported 2D and non-2D perovskite structures incorporating 86 amines, Wu et al. developed a supervised machine learning model to predict whether a given amine would form 2D perovskites or non-2D perovskites (Figure 4a).^82^ Moreover, the feature importance of amines is also extracted as guidance for 2D perovskites design. From the abstract of reported literatures, a natural language processing model also played a role by correlating the word “additives” with the actual functional intermediates and helped to find out the optimal additives to facilitate the perovskite crystallization process.^83^ Reviewing published studies to extract relevant information on materials of interest is straightforward. However, data sets of limited size (typically 10–1000 samples) are insufficient to support robust model training, providing only limited scope for algorithm optimization and increasing the risk of overfitting. Moreover, the tendency of the literature to report only successful experiments can lead to out-of-distribution (OOD) issues.^84^ OOD means models encounter data that are different from the training distribution. This is a well-known challenge in machine learning and must be carefully addressed when perovskite-related data sets are derived exclusively from reported successful studies.

**Figure 4:**
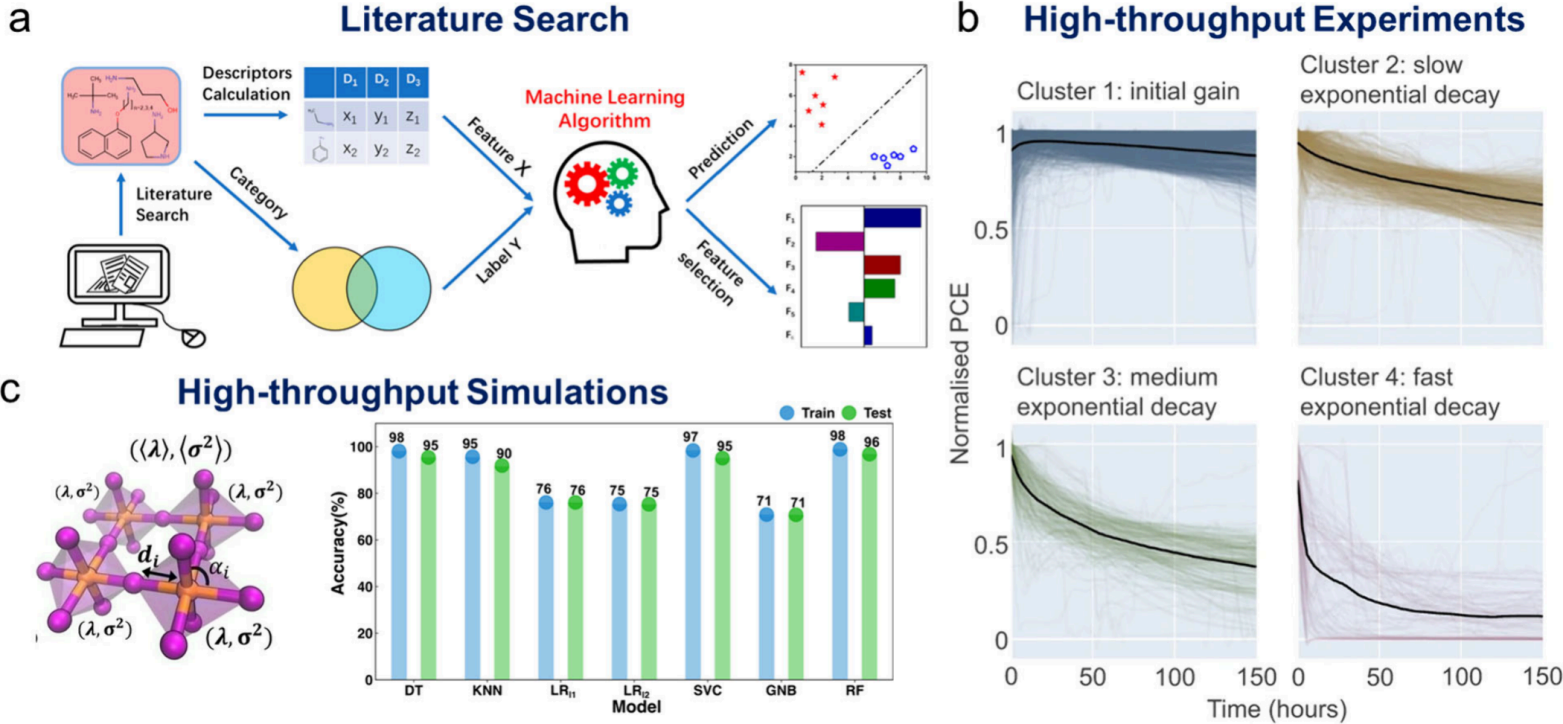
(a)­Workflow of predictive machine learning for 86 amines from a literature search. Reproduced from ref 82. Copyright 2021 American Chemical Society. (b) High-throughput aging systems with 2,245 MPPT aging curves collected and clustered into 4 types. Reproduced from ref 85. Available under a CC-BY 4.0 license. Copyright 2023 Springer Nature Hartono et al. (c) High-throughput MD simulations of 10,899 stable and unstable structures. Reproduced from ref 91. Available under a CC-BY 4.0 license. Copyright 2023 John Wiley and Sons Lin et al.

Beyond reported studies, data sets can be constructed through customized experimental characterization, which can be further accelerated by robotic automation. Without robotic platforms, experimental data collection is slow, yielding data set size in a scale comparable to or smaller than those from reported work. However, custom-built high-throughput experimental setups can generate data sets with thousands of samples. For example, a high-throughput aging system operated over 3 years by 22 researchers collected 2,245 maximum power point tracking (MPPT) aging curves of perovskite solar cells (Figure 4b).^85^ Although this approach provides unbiased and validated data tailoring important stability properties of PSCs, the process is still slow and costly, and the overall throughput remains limited.^86–88^

Besides literature and bespoke experimental strategies, high-throughput computational methods offer powerful tools to address data scarcity and provide the desired chemical data with higher structural diversity.^89,90^ For example, high-throughput DFT calculations have been employed to compute the binding energies of 168 pseudo halide anions in PSCs.^90^ Although DFT is accurate, it becomes expensive when it is applied in high-throughput workflows. Another high-throughput computational method is molecular dynamics (MD) simulation which is considered a “computational microscope”. Through algorithmic generation for 2D perovskites, 10,899 new organic cations have been generated through the combination of linkers, conjugated bodies, side chains and substituents (Figure 4d).^91^ High-throughput MD simulation characterized the stability of the as-formed 2D Pb–I perovskites incorporating the 10,899 cations based on the geometric bond-length and bond-angle deviance. The stabilization factors with the overall stability trend can be extracted and design rules for stable 2D ligands suggested. Large collections of molecules or materials are also available from well-known databases, such as PubChem, inorganic crystallographic structure database (ICSD), Materials Project, etc. For instance, Sumita et al. utilized 13,000 molecules from PubChem to train their ChemTS molecule generator based on a recurrent neural network model for the generation of molecules with desired wavelength.^92^ However, one should note that although the databases are large, specific properties of interest may not be available. Collecting reported data, conducting accelerated or automated experiments, employing high-throughput computational methods, and utilizing well-known databases are the four major approaches for applying machine learning to the study of perovskite hybrid materials. As shown in Table 1, we summarized the reported data set size in perovskite-focused chemistry research compiled from 39 published studies.^82,85,86,88,90–124^ In addition, extracted features used as inputs, machine learning models, evaluation metrics, and model outputs are also summarized. In Figure 5, we compared the data set size to that of established image classification and text-to-image generation data set in computer science.^125–127^ The median for perovskite-focused topic is only 229, far below the millions and billions of data set size in computer science. Constructing a material-based data set that includes the target properties can itself constitute a significant research endeavor.

Therefore, constructing a material-based data set that include the target properties can itself constitute a significant research endeavor.

**Table 1:** Summary of the Application of AI Tools in Perovskite-Focused Chemistry Research

References	Year	Data Set Size	Features	Machine Learning Models	Model Evaluation	Output
^93^	2020	250 Perovskite structures	Gibbs free energy of *OH and *O–*OH	Gaussian process regression (GPR), adaptive learning strategy	Root mean square error (RMSE) <0.5 eV	Overpotential (OER activity of ABO_3_-type perovskites)
^92^	2018	13000 Molecules (from PubChem)	Absorption wavelength	Monte Carlo tree search, recurrent neural network	86 out of 3200 have errors within 20 nm	New molecules
^86^	2019	50 Amines	Molecular descriptors	Logistic regression (LR), support vector machine (SVM), K-nearest neighbors (KNN), decision tree (DT), Gaussian Naïve Bayes (GNB)	Accuracy 86%	Amine post-treatment is compatible with perovskite or not
^94^	2020	184 PL spectra	Composition	GPR	RMSE = 0.08–0.1 eV	Bandgap, stability
^95^	2024	1000 PL images	Fluorescence intensity and texture morphology	SVM	Accuracy 92%	Tumor or normal
^96^	2024	27 Ammonium iodides	16 Feature descriptors	LR, Random forest (RF), gradient boosting (GB), support vector regression (SVR), neural network (NN), GPR, ensemble learning	Mean absolute error (MAE) 0.05–0.18	PCE
^97^	2024	15 SEM images	Grain boundaries	Convolutional neural network (CNN)	NA	PCE, FF, *J*_SC_, *V*_OC_
 ^98^	2019	333 Data points from publications	Composition	LR, KNN, SVR, RF, NN	RMSE 0.88% for bandgap	Bandgap, PCE
^99^	2022	100 from publications	Features like Eg, Rn, trap, size, TRPL	Ridge, PLSR, GPR, SVR	R^2^ 0.94	PCE, FF, *J*_SC_, *V*_OC_
 ^88^	2023	100 Solar cells from experiments	Cations, composition, annealing temperature	Multiple linear regression with expectation maximization	Standard error of estimation 0.21 for PCE	PCE, FF, *J*_SC_, *V*_OC_
 ^100^	2023	61 Experiments	Experimental parameter combinations	Bayesian optimization (BO)	NA	Optimal parameter set
^101^	2024	749 Data points	299 Unique perovskite compositions	LR, RF	RMSE/σ = 0.556 ± 0.056	Area specific resistance
^102^	2024	480 Drop-casting synthesis	Composition	Gated Gaussian process, BO	PCE 15–60% enhancements	PL, PCE
^103^	2025	160 Samples	Process parameters	GPR	Kullback–Leibler (KL) divergence between model updates fell to ∼2%	Weight of UV–vis, PL intensity, and PL imaging homogeneity
^123^	2023	100 Syntheses	Composition	GPR, NN, RF	Identified ∼ 95% of single-peak samples and about 44% of multipeak samples	Optimal precursor ratios
^104^	2024	850 Samples (XRF)	Measurement points from XRF, XRD, Ramen, ellipsometry	KNN, Lasso, kernel ridge regression (KRR), SVM, RF, multilayer perceptron, GPR	R^2^ 0.959 ± 0.006	Optimal stoichiometry of Mn, Co, Fe
^105^	2025	20000 Images	100 × 100-pixel image (flattened vector)	NN (ReLU activation function)	Accuracy 96%	Shape recognition
^106^	2025	33 Ammonium ligands	13 Structure features	RF, gradient boosting, XGBoost, LR	AUC = 0.88	Best ammonium ligands with low periodic transport barrier
^107^	2025	79 from synthesis, 287 include synthesis parameters	Molecular descriptors (encoding)	GPR, BO	Error ± 3 nm to ± 8 nm	PL Wavelength, fwhm, PLQY
^91^	2023	10848 Organic cations for RP 2D perovskites	Structures from MD simulations, nine molecular features	DT, KNN, LR, support vector classification (SVC), GNB, and RF	Accuracy 98%	2D perovskite is stable or not
^108^	2023	31 Double perovskites	Electronic structures from DFT	Tree-based models, RF, extra tree, gradient boosted regression (GBR), extreme gradient boosting	R^2^ 0.99 (training)/0.91 (test)	Substitution elements
^109^	2025	3239 for DJ 2D perovskites	Molecular features (encoding), electronic structures from DFT	LR, LASSO, Ridge, elastic net regression, SVR, RF, KNN	0.95 (training score)/0.95 (test score)	HOMO, LUMO
^110^	2025	3500 Double perovskites	Composition, atomic properties, 177 features	GBR	Precision 76%	Activity for OER
^111^	2017	298 Bimetallic alloy catalysts	13 Descriptors	Bootstrapped projected gradient descent	R^2^ 0.92, error 0.17 eV	Binding energy of CO
^112^	2020	515 2D perovskites	Structures from CIF	XGBoost	R^2^ 0.9005	Band gap
^113^	2025	10000 Organic cations for RP quasi-2D Perovskites	Morgan fingerprints, Chemprop embeddings, geometry	RF-Geometry, RF-Morgan, RF-Chemprop, and pretrained Chemprop models	Accuracy 97%	*n* = 3 Quasi-2D perovskite is stable or not
^114^	2022	229 Halide perovskite alloys	45 Descriptors	NN	Lattice constant RMSE 0.10Å	Lattice constant, decomposition energy, band gap, etc.
^115^	2022	2401 Perovskite structures	A-site and B-site cations, tolerance factors	RF	MAE 0.284–0.657 eV	Gibbs free energy
^116^	2024	60 Different perovskite compositions	Process parameters	BO	Improve PCE to 25.76% within 80 iterations	PCE
^117^	2022	100 Experimental conditions	Process parameters	BO, GP	10 conditions yielded PCE ≥17%	PCE
^82^	2021	86 Reported amine structures	21 Descriptors	LR, SVM, KNN, DT, GNB	Accuracy 82%	Forming 2D perovskite or not
^118^	2022	181 Reported PSCs	14 Features	LR, SVR, KNR, RFR, GBR	R^2^ 0.9172	PCE, FF, *J*_SC_, *V*_OC_
 ^119^	2018	635 Unmixed garnets	Ionic radii, electronegativities	NN	MAE 7–10 meV/atom	Formation energy of C_3_A_2_D_3_O_12_ garnets and ABO_3_ perovskites
^85^	2023	2245 MPPT decay curves of solar cells	Normalized PCE over time	Self-organizing map (SOM)-unsupervised clustering	Agree with the k-means clustering method	4 Degradation curve clusters
^120^	2018	167 Experimentally studied compositions	5 Features	SVC, regression, active learning	Accuracy: 77.5 ± 6.4%	Perovskite composition
^90^	2023	267 Pseudohalide anions	Four primary features	RF, LR	Accuracy 84%	High binding energy or low binding energy
^124^	2023	30 (+10) Crystal structures	Graph, composition	Message passing NN	Accuracy 88.2%–89.7%	Overpotentials
^122^	2018	108 Binary semiconductors	General chemical descriptors	KRR, LASSO, GBR	RMSE 0.34 eV	Bandgap
^123^	2024	3159 B-site alloyed perovskite structures	Graph, composition	Crystal graph CNN	Accuracy 96%	Bandgap, decomposition energy

**Figure 5:**
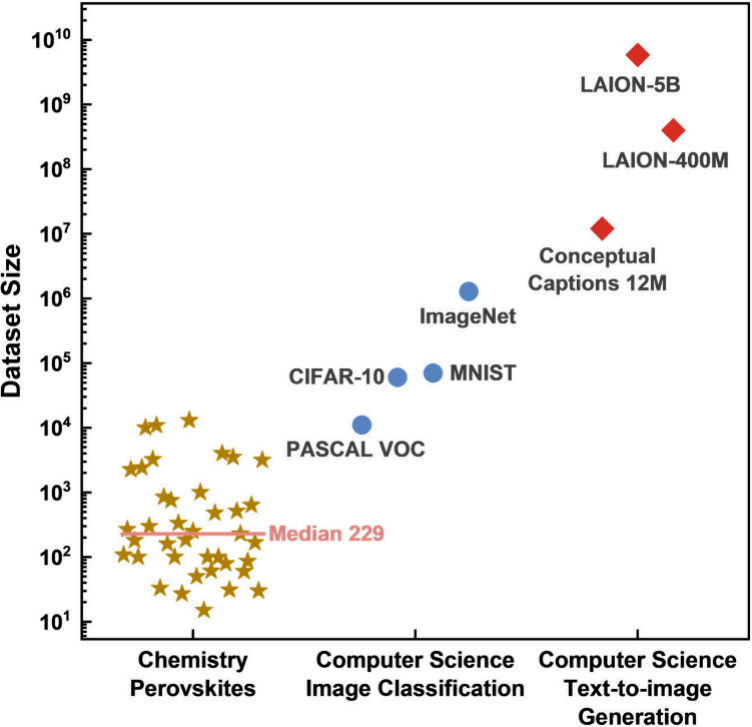
A comparison of data set sizes in perovskite-focused chemistry research (compiled from 39 published studies^82,85,86,88,90–124^ ) and widely used image classification and text-to-image generation data sets in computer science.^125–127^

With a sufficient data set in place, we can shift to machine learning model construction. A broad spectrum of algorithms, including linear regression, neural networks, Gaussian process regression, XGBoost, and support vector machines, have been applied. However, establishing baseline performance and comparing it with reference models are essential to substantiate improvements. Many existing studies present new application scenarios and directly apply off-the-shelf machine learning models, reporting only accuracy or error. Crucially, leveraging domain knowledge from chemistry and computer science to tailor models for material discovery is more important than merely demonstrating the application of machine learning techniques. Moreover, once the model is trained, large-scale virtual screening of materials (i.e., thousands of molecules) becomes technically straightforward. The central concern, therefore, is not the number of candidates screened but whether the model can accurately predict the properties of these candidates, which ultimately defines its practical value.

Broadly, the model of machine learning can be mainly classified into two categories: discriminative models (including classification and regression) and generative models. The latter one, direct creation of new materials with desired chemical properties, is an emerging research direction recently. A representative example is MatterGen, reported by Xie and co-workers, which adopted the most advanced diffusion model from computer science to generate inorganic materials with high accuracy and stability.^76^ However, one should note that training a generative model requires data sets that are several orders of magnitude larger than those used for discriminative tasks. As shown in Figure 5, compared to image classification, text-to-image generation has the data set expanded significantly. Although the final experimental validation has 20% differences, it will be compelling to see new materials generated with the desired electronic, mechanical, catalytic, and magnetic properties.

## Outlook

In summary, organic cations, through variations in dihedral angles, ammonium termini, and conjugated backbones, enable the design of a new family of low-dimensional perovskite materials. Enantiopure cations introduce chirality via secondary interactions to the inorganic framework, and the CISS effect highlights opportunities for spintronic applications. Machine learning further accelerates discovery by enabling efficient screening, prediction, and optimization.

OIMHPs provide a unique platform where inorganic lattices contribute mobility, conductivity, and thermal stability, while organic moieties impart novel functionality such as molecular conjugation, chirality, and processability. This duality enables the design of tailored hybrid semiconductors with functionalities, including solution-processable semiconductors with easy fabrication, molecular quantum wells, and tunable bandgap under pressure, that are inaccessible to purely inorganic or organic materials.

### Stability as a Foundation

Despite the growing number of reported OIMHP structures, systematic investigations of their stability remain scarce, yet such an understanding is crucial for achieving long device lifetimes. Establishing standardized protocols for evaluating the thermal, aqueous, and photostability of the OIMHPs is therefore imperative. Only with consistent evaluation frameworks could more feedback be provided to instruct rational hybrid structure engineering and molecular design. For example, is 2D perovskite with layered inorganic sheets capped by organic cations more stable than 1D perovskites with chain structures surrounded by organic cations? Are RP-phase 2D OIMHPs with monovalent cations and van der Waals interactions more stable than DJ-phase 2D OIMHPs with divalent cations? Furthermore, which types of hydrophobic units can improve aqueous stability and which structural units contribute to improved thermal or light stability? In addition to direct stability measurements, complementary approaches, such as electrochemical capacity measurements^128^ or mobile ion characterizations,^129^ should be developed to assess stability more efficiently and reduce the time required for long-term testing. Such benchmarks will guide the rational cation design and enable reliable device integration.

Developing a foundational model which encodes cost-effective yet information-rich structural representation tailored for organic–inorganic hybrids may redefine how OIMHPs are discovered.

### Chirality and Spin Functionality

Chiral OIMHPs open a new frontier in spin-selective optoelectronics and catalysis. The predominant strategy involves incorporation of chiral organic cations. Although several such cations have been reported for the synthesis of chiral OIMHPs, fundamental questions remain: How can one quantify the extent of chiral transfer from organic cations to inorganic frameworks? What structural descriptors of the organic components should be used to establish correlations between molecular structure and the resulting octahedral distortion and spin splitting? Expanding the library of chiral organic cations and obtaining more chiral OIMHP single crystals, potentially accelerated by AI-guided design, will be crucial for predicting promising candidate materials.

Beyond optical and spintronic demonstrations, exciting opportunities exist in spin-polarized electrocatalysis and photoelectrocatalysis. Chiral OIMHPs can manipulate electron spin, enabling spin-dependent electrochemistry to enhance the electrocatalytic performance. For instance, perovskite photoelectrodes can serve as spin polarizers to boost triplet O_2_ generation and thereby improve water-splitting efficiency.^130^ Spin polarization also offers the ability to regulate product selectivity in electrocatalytic reactions and to achieve enantioselective electro- or photoelectrosynthesis of chiral products.

One of the major challenges in spin-dependent electrochemistry lies in the limited stability of chiral OIMHPs under electrochemical conditions. Stabilizing photoelectrodes often requires a protective passivation layer to shield the moisture-sensitive perovskite while preserving strong spin polarization. Future research must focus on integrating structural stability with robust spin functionality to unlock the full potential of chiral OIMHPs.

### AI for Hybrid Materials

Machine learning offers powerful solutions to challenges that are difficult to accomplish experimentally, such as prediction of single-crystal structures. Learned from 50,000 crystal structures, a deep learning neural network can capture elemental distinctions, producing classification similar to the periodic table,^131^ yet applications to organic–inorganic hybrids remain limited. The reported known structures of OIMHPs are also much less for a machine to learn. Given that available data sets are typically several orders of magnitude smaller than those used in modern AI, such as the trillions of tokens for large language model training or the massive data sets in autonomous driving or image classification, the most promising method is to employ the large materials databases and develop high-throughput calculations to rationally tailor the data set of hybrid structures. Even modest predictive accuracy of single crystal structures would transform experimental design, while extending predictions to functional properties such as band edges, redox potentials, or *n* values of quasi-2D perovskites represents exciting directions.

Another major challenge is converting hybrid structural information to input vectors that a machine can interpret. Organic molecules involve chemical bonds between atoms, complex stereochemistry, rotational flexibility, chirality, and diverse functional groups, whereas inorganic materials contain periodic unit cells, space groups with varying symmetries, and atomic characteristics. The unique systems of OIMHPs which combine organic and inorganic components together present a greater level of complexity than organic molecules or inorganic lattices alone.

### The Road Ahead

Moving forward, the OIMHP research will benefit from synergies among conjugated hybrids, chiral materials, and AI-driven discovery. Addressing stability at the molecular level, quantifying chirality transfer, and exploiting spin polarization in electrochemical contexts are promising directions. AI offers a powerful means to accelerate the discovery of such unique properties by navigating and surpassing paths that would otherwise lead to failure. Success in these directions could establish OIMHPs as a paradigm-shifting class of materials for energy, optoelectronics, and quantum technologies.

## References

[1] Saparov B., Mitzi D. B. (2016). Organic-Inorganic Perovskites: Structural Versatility
for Functional Materials Design. Chem. Rev..

[2] Kagan C. R., Mitzi D. B., Dimitrakopoulos C. D. (1999). Organic-Inorganic Hybrid Materials
as Semiconducting Channels in Thin-Film Field-Effect Transistors. Science.

[3] Tremblay M. H., Boyington A., Rigin S., Jiang J., Bacsa J., Al Kurdi K., Khrustalev V. N., Pachter R., Timofeeva T. V., Jui N., Barlow S., Marder S. R. (2022). Hybrid Organic Lead Iodides: Role
of Organic Cation Structure in Obtaining 1D Chains of Face-Sharing
Octahedra vs 2D Perovskites. Chem. Mater..

[4] Zhao X., Liu T., Loo Y. L. (2022). Advancing 2D Perovskites for Efficient and Stable Solar
Cells: Challenges and Opportunities. Adv. Mater..

[5] Wang Z., Jingjing Q., Wang X., Zhang Z., Chen Y., Huang X., Huang W. (2018). Two-Dimensional Light-Emitting Materials:
Preparation, Properties and Applications. Chem.
Soc. Rev..

[6] Baek S., Shao W., Feng W., Tang Y., Lee Y. H., Loy J., Gunnarsson W. B., Yang H., Zhang Y., Faheem M. B., Kaswekar P. I., Atapattu H. R., Qin J., Coffey A. H., Park J. Y., Yang S. J., Yang Y., Zhu C., Wang K., Rand B. P., Dou L. (2024). Grain
Engineering for Efficient Near-Infrared Perovskite Light-Emitting
Diodes. Nat. Commun..

[7] Yu W., Li F., Yu L., Niazi M. R., Zou Y., Corzo D., Basu A., Ma C., Dey S., Tietze M. L., Buttner U., Wang X., Wang Z., Hedhili M. N., Guo C., Wu T., Amassian A. (2018). Single Crystal Hybrid Perovskite
Field-Effect Transistors. Nat. Commun..

[8] Kim Y. H., Zhai Y., Lu H., Pan X., Xiao C., Gaulding E. A., Harvey S. P., Berry J. J., Vardeny Z. V., Luther J. M., Beard M. C. (2021). Chiral-Induced Spin
Selectivity Enables
a Room-Temperature Spin Light-Emitting Diode. Science.

[9] Lu H., Wang J., Xiao C., Pan X., Chen X., Brunecky R., Berry J. J., Zhu K., Beard M. C., Vardeny Z. V. (2019). Spin-Dependent Charge Transport through 2D Chiral Hybrid
Lead-Iodide Perovskites. Sci. Adv..

[10] Blancon J.-C., Stier A. V., Tsai H., Nie W., Stoumpos C. C., Traoré B., Pedesseau L., Kepenekian M., Katsutani F., Noe G. T., Kono J., Tretiak S., Crooker S. A., Katan C., Kanatzidis M. G., Crochet J. J., Even J., Mohite A. D. (2018). Scaling Law for
Excitons in 2D Perovskite Quantum Wells. Nat.
Commun..

[11] Grätzel M. (2017). The Rise of
Highly Efficient and Stable Perovskite Solar Cells. Acc. Chem. Res..

[12] Gao P., Grätzel M., Nazeeruddin M. K. (2014). Organohalide Lead Perovskites for
Photovoltaic Applications. Energy Environ. Sci..

[13] Jeon N. J., Noh J. H., Yang W. S., Kim Y. C., Ryu S., Seo J., Seok S. Il. (2015). Compositional
Engineering of Perovskite Materials for
High-Performance Solar Cells. Nature.

[14] Kojima A., Teshima K., Shirai Y., Miyasaka T. (2009). Organometal Halide
Perovskites as Visible-Light Sensitizers for Photovoltaic Cells. J. Am. Chem. Soc..

[15] Kim H. S., Lee C. R., Im J. H., Lee K. B., Moehl T., Marchioro A., Moon S. J., Humphry-Baker R., Yum J. H., Moser J. E., Grätzel M., Park N. G. (2012). Lead Iodide Perovskite Sensitized
All-Solid-State Submicron
Thin Film Mesoscopic Solar Cell with Efficiency Exceeding 9%. Sci. Rep..

[16] Chung I., Lee B., He J., Chang R. P. H., Kanatzidis M. G. (2012). All-Solid-State
Dye-Sensitized Solar Cells with High Efficiency. Nature.

[17] Best Research-Cell Efficiency Chart. https://www.nrel.gov/pv/cell-efficiency.html.

[18] Miyata K., Atallah T. L., Zhu X.-Y. (2017). Lead Halide
Perovskites: Crystal-Liquid
Duality, Phonon Glass Electron Crystals, and Large Polaron Formation. Sci. Adv..

[19] Boyd C. C., Cheacharoen R., Leijtens T., McGehee M. D. (2019). Understanding Degradation
Mechanisms and Improving Stability of Perovskite Photovoltaics. Chem. Rev..

[20] Sun J., Wang K., Ma K., Park J. Y., Lin Z. Y., Savoie B. M., Dou L. (2023). Emerging Two-Dimensional
Organic
Semiconductor-Incorporated Perovskites–A Fascinating Family
of Hybrid Electronic Materials. J. Am. Chem.
Soc..

[21] Li X., Yang J., Song Z., Chen R., Ma L., Li H., Jia J., Meng J., Li X., Yi M., Sun X. (2018). Naphthalene Diimide Ammonium Directed Single-Crystalline Perovskites
with “Atypical” Ambipolar Charge Transport Signatures
in Two-Dimensional Limit. ACS Appl. Energy.
Mater..

[22] Billing D. G., Lemmerer A. (2003). Bis­[(S)-β-Phenethylammonium] Tribromoplumbate­(II). Acta Crystallogr. Sect. E: Struct. Rep..

[23] Chen W., Shi Y., Chen J., Ma P., Fang Z., Ye D., Lu Y., Yuan Y., Zhao J., Xiao Z. (2021). Polymerized Hybrid
Perovskites with Enhanced Stability, Flexibility, and Lattice Rigidity. Adv. Mater..

[24] Wang Y., Yan D., Wang L., Wang D., Tang B. Z. (2021). Aggregation-Induced
Emission Luminogens Sensitized Quasi-2D Hybrid Perovskites with Unique
Photoluminescence and High Stability for Fabricating White Light-Emitting
Diodes. Adv. Sci..

[25] Choi H. S., Lin J., Wang G., Wong W. P. D., Park I.-H., Lin F., Yin J., Leng K., Lin J., Loh K. P. (2024). Molecularly Thin,
Two-Dimensional All-Organic Perovskites. Science.

[26] Folgueras M. C., Jiang Y., Jin J., Yang P. (2023). High-Entropy Halide
Perovskite Single Crystals Stabilized by Mild Chemistry. Nature.

[27] Zhang S., Jin L., Lu Y., Zhang L., Yang J., Zhao Q., Sun D., Thompson J. J. P., Yuan B., Ma K., Akriti, Park J. Y., Lee Y. H., Wei Z., Finkenauer B. P., Blach D. D., Kumar S., Peng H., Mannodi-Kanakkithodi A., Yu Y., Malic E., Lu G., Dou L., Huang L. (2024). Moiré
Superlattices in Twisted Two-Dimensional Halide Perovskites. Nat. Mater..

[28] Gao M., Park Y., Jin J., Chen P. C., Devyldere H., Yang Y., Song C., Lin Z., Zhao Q., Siron M., Scott M. C., Limmer D. T., Yang P. (2023). Direct Observation
of Transient Structural Dynamics of Atomically Thin Halide Perovskite
Nanowires. J. Am. Chem. Soc..

[29] Song M., Zhao B., Li B., Wang K., Jiang Y., Jia G., Zhao X., Yu B., Li Y., Yang F. (2025). Synthesis
of Single-Unit-Cell-Thick Perovskites by Liquid-Phase Confined Assembly
for High-Performance Ultrastable X-Ray Detectors. Nat. Syn..

[30] Gao Y., Shi E., Deng S., Shiring S. B., Snaider J. M., Liang C., Yuan B., Song R., Janke S. M., Liebman-Peláez A., Yoo P., Zeller M., Boudouris B. W., Liao P., Zhu C., Blum V., Yu Y., Savoie B. M., Huang L., Dou L. (2019). Molecular Engineering of Organic–Inorganic Hybrid Perovskites
Quantum Wells. Nat. Chem..

[31] Mitzi D. B., Chondroudis K., Kagan C. R. (1999). Design, Structure, and Optical Properties
of Organic-Inorganic Perovskites Containing an Oligothiophene Chromophore. Inorg. Chem..

[32] Li B., Liu Q., Gong J., Li S., Zhang C., Gao D., Chen Z., Li Z., Wu X., Zhao D., Yu Z., Li X., Wang Y., Lu H., Zeng X. C., Zhu Z. (2024). Harnessing Strong Aromatic Conjugation
in Low-Dimensional Perovskite
Heterojunctions for High-Performance Photovoltaic Devices. Nat. Commun..

[33] Xue J., Wang R., Chen X., Yao C., Jin X., Wang K. L., Huang W., Huang T., Zhao Y., Zhai Y., Meng D., Tan S., Liu R., Wang Z. K., Zhu C., Zhu K., Beard M. C., Yan Y., Yang Y. (2021). Reconfiguring the Band-Edge States of Photovoltaic
Perovskites by Conjugated Organic Cations. Science.

[34] Ma K., Atapattu H. R., Zhao Q., Gao Y., Finkenauer B. P., Wang K., Chen K., Park S. M., Coffey A. H., Zhu C., Huang L., Graham K. R., Mei J., Dou L. (2021). Multifunctional
Conjugated Ligand Engineering for Stable and Efficient Perovskite
Solar Cells. Adv. Mater..

[35] Ma K., Sun J., Atapattu R. H., Larson W. B., Yang H., Sun D., Chen K., Wang K., Lee Y., Tang Y., Bhoopalam A., Huang L., Graham R. K., Mei J., Dou L. (2023). Holistic Energy
Landscape Management in 2D/3D Heterojunction via
Molecular Engineering for Efficient Perovskite Solar Cells. Sci. Adv..

[36] Chandra
Patra B., Wan R., Moore C. E., Wu Y. (2025). Impact of
Dihedral Angle in Conjugated Organic Cation on the Structures and
Properties of Organic-Inorganic Lead Iodides. Chem. Eur. J..

[37] Wang K., Lin Z. Y., Zhang Z., Jin L., Ma K., Coffey A. H., Atapattu H. R., Gao Y., Park J. Y., Wei Z., Finkenauer B. P., Zhu C., Meng X., Chowdhury S. N., Chen Z., Terlier T., Do T. H., Yao Y., Graham K. R., Boltasseva A., Guo T. F., Huang L., Gao H., Savoie B. M., Dou L. (2023). Suppressing Phase Disproportionation
in Quasi-2D Perovskite Light-Emitting Diodes. Nat. Commun..

[38] Wan R., Lyu R., Moore C. E., Wu Y. (2023). Toward Understanding
the Composition-Structure
Relationship of Hybrid Organic Lead Iodide Compounds: Impact from
Secondary Structures of Organic Cations. J.
Phys. Chem. C.

[39] Passarelli J. V., Fairfield D. J., Sather N. A., Hendricks M. P., Sai H., Stern C. L., Stupp S. I. (2018). Enhanced Out-of-Plane Conductivity
and Photovoltaic Performance in n = 1 Layered Perovskites through
Organic Cation Design. J. Am. Chem. Soc..

[40] Sun J., Ma K., Lin Z. Y., Tang Y., Varadharajan D., Chen A. X., Atapattu H. R., Lee Y. H., Chen K., Boudouris B. W., Graham K. R., Lipomi D. J., Mei J., Savoie B. M., Dou L. (2023). Tailoring Molecular-Scale Contact
at the Perovskite/Polymeric Hole-Transporting Material Interface for
Efficient Solar Cells. Adv. Mater..

[41] Gao Y., Wei Z., Yoo P., Shi E., Zeller M., Zhu C., Liao P., Dou L. (2019). Highly Stable
Lead-Free Perovskite
Field-Effect Transistors Incorporating Linear Π-Conjugated Organic
Ligands. J. Am. Chem. Soc..

[42] Lyu R., Cui Z., Elgin J., Co A. C., Wu Y. (2023). Photoelectrochemistry
of Methylviologen Lead Iodide: Achieving Stability inside a Polar
Solvent. J. Phys. Chem. C.

[43] Liu C., Yang Y., Chen H., Spanopoulos I., Bati A. S. R., Gilley I. W., Chen J., Maxwell A., Vishal B., Reynolds R. P., Wiggins T. E., Wang Z., Huang C., Fletcher J., Liu Y., Chen L. X., De Wolf S., Chen B., Zheng D., Marks T. J., Facchetti A., Sargent E. H., Kanatzidis M. G. (2024). Two-Dimensional
Perovskitoids Enhance Stability in Perovskite Solar Cells. Nature.

[44] Zhao R., Sabatini R. P., Zhu T., Wang S., Morteza
Najjarian A., Johnston A., Lough A. J., Hoogland S., Sargent E. H., Seferos D. S. (2021). Rigid Conjugated Diamine Templates
for Stable Dion-Jacobson-Type Two-Dimensional Perovskites. J. Am. Chem. Soc..

[45] Shi E., Yuan B., Shiring S. B., Gao Y., Akriti, Guo Y., Su C., Lai M., Yang P., Kong J., Savoie B. M., Yu Y., Dou L. (2020). Two-Dimensional Halide
Perovskite Lateral Epitaxial Heterostructures. Nature.

[46] Akriti, Zhang S., Lin Z. Y., Shi E., Finkenauer B. P., Gao Y., Pistone A. J., Ma K., Savoie B. M., Dou L. (2021). Quantifying
Anionic Diffusion in
2D Halide Perovskite Lateral Heterostructures. Adv. Mater..

[47] Wang M., Shi Z., Fei C., Deng Z. J. D., Yang G., Dunfield S. P., Fenning D. P., Huang J. (2023). Ammonium Cations with High PKa in
Perovskite Solar Cells for Improved High-Temperature Photostability. Nat. Energy.

[48] Yang Y., Chen H., Liu C., Xu J., Huang C., Malliakas C. D., Wan H., Bati A. S. R., Wang Z., Reynolds R. P., Gilley I. W., Kitade S., Wiggins T. E., Zeiske S., Suragtkhuu S., Batmunkh M., Chen L. X., Chen B., Kanatzidis M. G., Sargent E. H. (2024). Amidination of Ligands
for Chemical and Field-Effect Passivation Stabilizes Perovskite Solar
Cells. Science.

[49] Yang T., Ma C., Cai W., Wang S., Wu Y., Feng J., Wu N., Li H., Huang W., Ding Z., Gao L., Liu S. F., Zhao K. (2023). Amidino-Based Dion-Jacobson 2D Perovskite
for Efficient and Stable 2D/3D Heterostructure Perovskite Solar Cells. Joule.

[50] Sun J., Wang J., Yao Z., Bi L., Ji X., Wang J., Huang X., Liu M., Liu K., Lin F. R., Kan B., Fu Q., Jen A. K. (2025). Molecular
Engineering of Terminus, Conjugation, and Energetics for Thermally
Stable Inverted Perovskite Solar Cells. J. Am.
Chem. Soc..

[51] Liu C., Yang Y., Fletcher J. D., Liu A., Gilley I. W., Musgrave C. B., Wang Z., Zhu H., Chen H., Reynolds R. P., Ding B., Ding Y., Zhang X., Skackauskaite R., Wan H., Zeng L., Bati A. S. R., Shibayama N., Getautis V., Chen B., Rakstys K., Dyson P. J., Kanatzidis M. G., Sargent E. H., Nazeeruddin M. K. (2025). Cation
Interdiffusion Control for 2D/3D Heterostructure Formation and Stabilization
in Inorganic Perovskite Solar Modules. Nat.
Energy.

[52] Song Y., Zhou Y., Chen C., Fan K., Wang Z., Guo Y., Chen Z., Mao L., Yin J., Chow P. C. Y. (2025). Structure
– Emission Property Relationship of Bilayer 2D Hybrid Perovskites. J. Am. Chem. Soc..

[53] Sun J., Penukula S., Li M., Hosseinzade M. R., Tang Y., Dou L., Rolston N. (2024). Mechanical
and Ionic
Characterization for Organic Semiconductor-Incorporated Perovskites
for Stable 2D/3D Heterostructure Perovskite Solar Cells. Small.

[54] Ahn J., Lee E., Tan J., Yang W., Kim B., Moon J. (2017). A New Class
of Chiral Semiconductors: Chiral-Organic-Molecule-Incorporating Organic–Inorganic
Hybrid Perovskites. Mater. Horiz..

[55] Long G., Sabatini R., Saidaminov M. I., Lakhwani G., Rasmita A., Liu X., Sargent E. H., Gao W. (2020). Chiral-Perovskite Optoelectronics. Nat. Rev.
Mater..

[56] Pietropaolo A., Mattoni A., Pica G., Fortino M., Schifino G., Grancini G. (2022). Rationalizing the Design and Implementation
of Chiral
Hybrid Perovskites. Chem..

[57] Ma S., Ahn J., Moon J. (2021). Chiral Perovskites
for Next-Generation Photonics: From
Chirality Transfer to Chiroptical Activity. Adv. Mater..

[58] Yao J., Wang Z., Huang Y., Xue J., Zhang D., Chen J., Chen X., Dong S. C., Lu H. (2024). Efficient
Green Spin Light-Emitting Diodes Enabled by Ultrafast Energy- and
Spin-Funneling in Chiral Perovskites. J. Am.
Chem. Soc..

[59] Duan T., You S., Chen M., Yu W., Li Y., Guo P., Berry J. J., Luther J. M., Zhu K., Zhou Y. (2024). Chiral-Structured
Heterointerfaces Enable Durable Perovskite Solar Cells. Science.

[60] Yang C. H., Xiao S. B., Xiao H., Xu L. J., Chen Z. N. (2023). Efficient
Red-Emissive Circularly Polarized Electroluminescence Enabled by Quasi-2D
Perovskite with Chiral Spacer Cation. ACS Nano.

[61] Billing D. G., Lemmerer A. (2006). Synthesis and Crystal Structures of Inorganic-Organic
Hybrids Incorporating an Aromatic Amine with a Chiral Functional Group. CrystEngComm.

[62] Yang C. K., Chen W. N., Ding Y. T., Wang J., Rao Y., Liao W. Q., Tang Y. Y., Li P. F., Wang Z. X., Xiong R. G. (2019). The First 2D Homochiral Lead Iodide Perovskite Ferroelectrics:
[R- and S-1-(4-Chlorophenyl)­Ethylammonium]­2PbI4. Adv. Mater..

[63] Wang Z., Zhao G., Zhang H., Zhou H., Tang Z. (2025). Structural
Design and Applications of Chiral Perovskites. Energy Mater. Adv..

[64] Ray K., Ananthavel S. P., Waldeck D. H., Naaman R. (1999). Asymmetric Scattering
of Polarized Electrons by Organized Organic Films of Chiral Molecules. Science.

[65] Hautzinger M. P., Pan X., Hayden S. C., Ye J. Y., Jiang Q., Wilson M. J., Phillips A. J., Dong Y., Raulerson E. K., Leahy I. A., Jiang C.-S., Blackburn J. L., Luther J. M., Lu Y., Jungjohann K., Vardeny Z. V., Berry J. J., Alberi K., Beard M. C. (2024). Room-Temperature
Spin Injection across a Chiral Perovskite/III–V Interface. Nature.

[66] Jang G., Jo D. Y., Ma S., Lee J., Son J., Lee C. U., Jeong W., Yang S., Park J. H., Yang H., Moon J. (2024). Core–Shell Perovskite
Quantum
Dots for Highly Selective Room-Temperature Spin Light-Emitting Diodes. Adv. Mater..

[67] Tang Z., Guloy A. M. (1999). A Methylviologen Lead­(II) Iodide: Novel [PbI3-]∞
Chains with Mixed Octahedral and Trigonal Prismatic Coordination. J. Am. Chem. Soc..

[68] Fujisawa J. I., Ishihara T. (2004). Charge-Transfer Transitions
between Wires and Spacers
in an Inorganic-Organic Quasi-One-Dimensional Crystal Methylviologen
Lead Iodide. Phys. Rev. B Condens. Matter.

[69] Lyu R., Wan R., Moore C. E., Wu Y. (2024). Chiral Viologen-Derived Water-Stable
Small Band Gap Lead Halides: Synthesis, Characterization, and Optical
Properties. Inorg. Chem..

[70] Wan R., Yin M., Wang T. H., Moore C. E., Wu Y. (2025). Chiral Methylbenzylpyridinium-Based
Organic-Inorganic Lead Halides for Water-Resistant Photoluminescence
Materials. Inorg. Chem..

[71] Jana M. K., Song R., Liu H., Khanal D. R., Janke S. M., Zhao R., Liu C., Valy Vardeny Z., Blum V., Mitzi D. B. (2020). Organic-to-Inorganic
Structural Chirality
Transfer in a 2D Hybrid Perovskite and Impact on Rashba-Dresselhaus
Spin-Orbit Coupling. Nat. Commun..

[72] Jana M. K., Song R., Xie Y., Zhao R., Sercel P. C., Blum V., Mitzi D. B. (2021). Structural
Descriptor for Enhanced
Spin-Splitting in 2D Hybrid Perovskites. Nat.
Commun..

[73] Lu D., Zhao Z., Xiang X., Li T., Geng Y., Wu M., Li Y., Xu S., Zhang C., Gao Z., Shen J.- W., Liang L., Fan K., Yao Z., Zhang L. (2025). A Smart Framework to Design Membranes for Organic Micropollutants
Removal. Nat. Sustain..

[74] Myung C. W., Hajibabaei A., Cha J. H., Ha M., Kim J., Kim K. S. (2022). Challenges,
Opportunities, and Prospects in Metal Halide
Perovskites from Theoretical and Machine Learning Perspectives. Adv. Energy. Mater..

[75] Ma J., Cao B., Dong S., Tian Y., Wang M., Xiong J., Sun S. (2024). MLMD: A Programming-Free
AI Platform to Predict and Design Materials. NPJ. Comput. Mater..

[76] Zeni C., Pinsler R., Zügner D., Fowler A., Horton M., Fu X., Shysheya S., Crabbé J., Sun L., Smith J., Nguyen B., Schulz H., Lewis S., Huang C.-W., Lu Z., Zhou Y., Yang H., Hao H., Li J., Tomioka R., Xie T. (2025). MatterGen: A Generative
Model for Inorganic Materials Design. Nature.

[77] Zhao Q., Savoie B. M. (2023). Deep Reaction Network Exploration of Glucose Pyrolysis. Proc. Natl. Acad. Sci. U. S. A..

[78] Tao Q., Xu P., Li M., Lu W. (2021). Machine Learning for Perovskite Materials
Design and Discovery. NPJ. Comput. Mater..

[79] Ding R., Liu J., Hua K., Wang X., Zhang X., Shao M., Chen Y., Chen J. (2025). Leveraging Data Mining, Active Learning,
and Domain Adaptation for Efficient Discovery of Advanced Oxygen Evolution
Electrocatalysts. Sci. Adv..

[80] Vamathevan J., Clark D., Czodrowski P., Dunham I., Ferran E., Lee G., Li B., Madabhushi A., Shah P., Spitzer M., Zhao S. (2019). Applications
of Machine Learning in Drug Discovery and Development. Nat. Rev. Drug Discovery.

[81] Roumeliotis K. I., Tselikas N. D. (2023). ChatGPT and Open-AI
Models: A Preliminary Review. Future Internet.

[82] Lyu R., Moore C. E., Liu T., Yu Y., Wu Y. (2021). Predictive
Design Model for Low-Dimensional Organic-Inorganic Halide Perovskites
Assisted by Machine Learning. J. Am. Chem. Soc..

[83] Ge J., Huang Y., Chen X., Wang Y., Zeng H., Tao Y., Zeng Z., Zhang L., Zhang L., Lu X., Tsang S. W., You J., Jen A. K. Y., Liu S. F. (2025). An Intermediate-Aided
Perovskite Phase Purification for High-Performance Solar Cells. J. Am. Chem. Soc..

[84] Cui P., Wang J. (2022). Out-of-Distribution
(OOD) Detection Based on Deep Learning: A Review. Electronics (Basel).

[85] Hartono N. T. P., Köbler H., Graniero P., Khenkin M., Schlatmann R., Ulbrich C., Abate A. (2023). Stability Follows Efficiency
Based on the Analysis of a Large Perovskite Solar Cells Ageing Dataset. Nat. Commun..

[86] Yu Y., Tan X., Ning S., Wu Y. (2019). Machine Learning for
Understanding
Compatibility of Organic-Inorganic Hybrid Perovskites with Post-Treatment
Amines. ACS Energy Lett..

[87] Sun S., Hartono N. T. P., Ren Z. D., Marius I., Sun S., Hartono N. T. P., Ren Z. D., Oviedo F., Buscemi A. M., Layurova M., Chen D. X., Ogunfunmi T., Thapa J., Ramasamy S., Settens C., Decost B. L., Kusne A. G., Liu Z., Tian S. I. P., Peters I. M. (2019). Accelerated Development of Perovskite-
Inspired Materials via High-Throughput
Synthesis and Machine-Learning Diagnosis Accelerated Development of
Perovskite- Inspired Materials via High-Throughput Synthesis and Machine-Learning
Diagnosis. Joule.

[88] Meftahi N., Surmiak M. A., Fürer S. O., Rietwyk K. J., Lu J., Raga S. R., Evans C., Michalska M., Deng H., McMeekin D. P., Alan T., Vak D., Chesman A. S. R., Christofferson A. J., Winkler D. A., Bach U., Russo S. P. (2023). Machine Learning
Enhanced High-Throughput Fabrication
and Optimization of Quasi-2D Ruddlesden–Popper Perovskite Solar
Cells. Adv. Energy Mater..

[89] Kim H. W., Han J. H., Ko H., Samanta T., Lee D. G., Jeon D. W., Kim W., Chung Y.-C., Im W. B., Cho S. B. (2023). High-Throughput
Screening on Halide Perovskite Derivatives
and Rational Design of Cs3LuCl6. ACS Energy
Lett..

[90] Xu J., Chen H., Grater L., Liu C., Yang Y., Teale S., Maxwell A., Mahesh S., Wan H., Chang Y., Chen B., Rehl B., Park S. M., Kanatzidis M. G., Sargent E. H. (2023). Anion Optimization for Bifunctional
Surface Passivation in Perovskite Solar Cells. Nat. Mater..

[91] Lin Z.-Y., Sun J., Shiring S. B., Dou L., Savoie B. M. (2023). Design Rules for
Two-Dimensional Organic Semiconductor-Incorporated Perovskites (OSiP)
Gleaned from Thousands of Simulated Structures. Angew. Chem., Int. Ed..

[92] Sumita M., Yang X., Ishihara S., Tamura R., Tsuda K. (2018). Hunting for
Organic Molecules with Artificial Intelligence: Molecules Optimized
for Desired Excitation Energies. ACS Cent. Sci..

[93] Li Z., Achenie L. E. K., Xin H. (2020). An Adaptive Machine Learning Strategy
for Accelerating Discovery of Perovskite Electrocatalysts. ACS Catal..

[94] Higgins K., Valleti S. M., Ziatdinov M., Kalinin S. V., Ahmadi M. (2020). Chemical Robotics
Enabled Exploration of Stability in Multicomponent Lead Halide Perovskites
via Machine Learning. ACS Energy Lett..

[95] Chi J., Xue Y., Zhou Y., Han T., Ning B., Cheng L., Xie H., Wang H., Wang W., Meng Q., Fan K., Gong F., Fan J., Jiang N., Liu Z., Pan K., Sun H., Zhang J., Zheng Q., Wang J., Su M., Song Y. (2024). Perovskite Probe-Based Machine Learning Imaging Model
for Rapid Pathologic Diagnosis of Cancers. ACS
Nano.

[96] Mai Y., Tang J., Meng H., Li X., Liu M., Chen Z., Zhang P., Li S. (2024). Machine Learning-Based
Screening of Two-Dimensional Perovskite Organic Spacers. Adv. Compos. Hybrid Mater..

[97] Li X., Mai Y., Meng H., Bi H., Ng C. H., Teo S. H., Lan C., Zhang P., Li S. (2024). Machine Learning Quantification of
Grain Boundary Defects for High Efficiency Perovskite Solar Cells. Adv. Compos. Hybrid Mater..

[98] Li J., Pradhan B., Gaur S., Thomas J. (2019). Predictions and Strategies
Learned from Machine Learning to Develop High-Performing Perovskite
Solar Cells. Adv. Energy Mater..

[99] Hu Y., Hu X., Zhang L., Zheng T., You J., Jia B., Ma Y., Du X., Zhang L., Wang J., Che B., Chen T., Liu S. F. (2022). Machine-Learning Modeling for Ultra-Stable
High-Efficiency Perovskite Solar Cells. Adv.
Energy Mater..

[100] Zhang J., Liu B., Liu Z., Wu J., Arnold S., Shi H., Osterrieder T., Hauch J. A., Wu Z., Luo J., Wagner J., Berger C. G., Stubhan T., Schmitt F., Zhang K., Sytnyk M., Heumueller T., Sutter-Fella C. M., Peters I. M., Zhao Y., Brabec C. J. (2023). Optimizing Perovskite
Thin-Film Parameter Spaces with Machine Learning-Guided Robotic Platform
for High-Performance Perovskite Solar Cells. Adv. Energy Mater..

[101] Jacobs R., Liu J., Abernathy H., Morgan D. (2024). Machine Learning Design of Perovskite Catalytic Properties. Adv. Energy Mater..

[102] Um M., Sanchez S. L., Song H., Lawrie B. J., Ahn H., Kalinin S. V., Liu Y., Choi H., Yang J., Ahmadi M. (2025). Tailoring Molecular
Space to Navigate Phase Complexity
in Cs-Based Quasi-2D Perovskites via Gated-Gaussian-Driven High-Throughput
Discovery. Adv. Energy Mater..

[103] Halder A., Alghalayini M. B., Cheng S., Thalanki N., Nguyen T. M., Hering A. R., Lee D. K., Arnold S., Leite M. S., Barnard E., Razumtcev A., Wall M., Gashi A., Liu Y. R., Noack M. M., Sun S., Sutter-Fella C. M. (2025). AI-Driven
Robot Enables Synthesis-Property
Relation Prediction for Metal Halide Perovskites in Humid Atmosphere. Adv. Energy Mater..

[104] Bozal-Ginesta C., Sirvent J., Cordaro G., Fearn S., Pablo-García S., Chiabrera F., Choi C., Laa L., Núñez M., Cavallaro A., Buzi F., Aguadero A., Dezanneau G., Kilner J., Morata A., Baiutti F., Aspuru-Guzik A., Tarancón A. (2024). Performance Prediction of High-Entropy
Perovskites La0.8Sr0.2MnxCoyFezO3 with Automated High-Throughput Characterization
of Combinatorial Libraries and Machine Learning. Adv. Mater..

[105] Opala A., Tyszka K., Kędziora M., Furman M., Rahmani A., Świerczewski S., Ekielski M., Szerling A., Matuszewski M., Piętka B. (2025). Perovskite Microwires for Room Temperature Exciton-Polariton
Neural Network. Adv. Mater..

[106] Yan G., Tang H., Shen Y., Han L., Han Q. (2025). AI-Generated
Ammonium Ligands for High-Efficiency and Stable 2D/3D Heterojunction
Perovskite Solar Cells. Adv. Mater..

[107] Henke N. A., Luber L., Kouroudis I., Paul J., Schuhbeck A., Rescher L. M., Lorenzen T., Mayer V., Müller-Caspary K., Nickel B., Gagliardi A., Urban A. S. (2026). Synthesizer: Chemistry-Aware Machine
Learning for Precision Control of Nanocrystal Growth. Adv. Mater..

[108] Li X., Mai H., Lu J., Wen X., Le T. C., Russo S. P., Winkler D. A., Chen D., Caruso R. A. (2023). Rational
Atom Substitution to Obtain Efficient, Lead-Free Photocatalytic Perovskites
Assisted by Machine Learning and DFT Calculations. Angew. Chem., Int. Ed..

[109] Lyu, Y. ; Zhou, Y. ; Zhang, Y. ; Yang, Y. ; Zou, B. ; Weng, Q. ; Xie, T. ; Cazorla, C. ; Hao, J. ; Yin, J. ; Wu, T. Fingerprinting Organic Molecules for the Inverse Design of Two-Dimensional Hybrid Perovskites with Target Energetics. arXiv preprint 2025, 2509.25728. 10.1126/sciadv.aeb4144 PMC13089347 41996511

[110] Mok D. H., Lim J., Chang H., Shin J., Jang J., Sim U., Gu G. H., Back S. (2025). Machine Learning
High-Throughput Screening of Double Perovskites for Enhanced Acidic
Oxygen Evolution. Chem. Eng. J..

[111] Pankajakshan P., Sanyal S., De Noord O. E., Bhattacharya I., Bhattacharyya A., Waghmare U. (2017). Machine Learning and
Statistical
Analysis for Materials Science: Stability and Transferability of Fingerprint
Descriptors and Chemical Insights. Chem. Mater..

[112] Marchenko E. I., Fateev S. A., Petrov A. A., Korolev V. V., Mitrofanov A., Petrov A. V., Goodilin E. A., Tarasov A. B. (2020). Database
of Two-Dimensional Hybrid Perovskite Materials: Open-Access Collection
of Crystal Structures, Band Gaps, and Atomic Partial Charges Predicted
by Machine Learning. Chem. Mater..

[113] Nian, Z. ; Savoie, B. M. Design of Hybrid Quasi-2D Perovskite Ligands to Improve Stability. ChemRxiv, 2025. 10.26434/chemrxiv-2025-8swnn.

[114] Mannodi-Kanakkithodi A., Chan M. K. Y. (2022). Data-Driven Design
of Novel Halide
Perovskite Alloys. Energy Environ. Sci..

[115] Wang X., Gao Y., Krzystowczyk E., Iftikhar S., Dou J., Cai R., Wang H., Ruan C., Ye S., Li F. (2022). High-Throughput
Oxygen
Chemical Potential Engineering of Perovskite Oxides for Chemical Looping
Applications. Energy Environ. Sci..

[116] Zhang B., Zeng H., Yin H., Zheng D., Wan Z., Jia C., Stuyver T., Luo J., Pauporte T. (2024). Combining
Component Screening, Machine Learning and Molecular Engineering for
the Design of High-Performance Inverted Perovskite Solar Cells. Energy Environ. Sci..

[117] Liu Z., Rolston N., Flick A. C., Colburn T. W., Ren Z., Dauskardt R. H., Buonassisi T. (2022). Machine Learning with Knowledge Constraints
for Process Optimization of Open-Air Perovskite Solar Cell Manufacturing
Machine Learning with Knowledge Constraints for Process Optimization
of Open-Air Perovskite Solar Cell Manufacturing. Joule.

[118] Cai X., Liu F., Yu A., Qin J., Hatamvand M., Ahmed I., Luo J., Zhang Y., Zhang H., Zhan Y. (2022). Data-Driven Design of High-Performance
MASnxPb1-XI3 Perovskite Materials
by Machine Learning and Experimental Realization. Light Sci. Appl..

[119] Ye W., Chen C., Wang Z., Chu I. H., Ong S. P. (2018). Deep Neural
Networks for Accurate Predictions of Crystal Stability. Nat. Commun..

[120] Balachandran P. V., Kowalski B., Sehirlioglu A., Lookman T. (2018). Experimental Search for High-Temperature Ferroelectric
Perovskites Guided by Two-Step Machine Learning. Nat. Commun..

[121] Zhang Y., Ling C. (2018). A Strategy to Apply Machine Learning
to Small Datasets in Materials Science. NPJ.
Comput. Mater..

[122] Kim J. S., Noh J., Im J. (2024). Machine Learning-Enabled
Chemical Space Exploration of All-Inorganic Perovskites for Photovoltaics. NPJ. Comput. Mater..

[123] Lampe C., Kouroudis I., Harth M., Martin S., Gagliardi A., Urban A. S. (2023). Rapid Data-Efficient Optimization
of Perovskite Nanocrystal Syntheses through Machine Learning Algorithm
Fusion. Adv. Mater..

[124] Moon J., Beker W., Siek M., Kim J., Lee H. S., Hyeon T., Grzybowski B. A. (2024). Active
Learning Guides Discovery of a Champion Four-Metal Perovskite Oxide
for Oxygen Evolution Electrocatalysis. Nat.
Mater..

[125] Brigato L., Barz B., Iocchi L., Denzler J. (2022). Image Classification
With Small Datasets: Overview and Benchmark. IEEE Access.

[126] Sharma, P. ; Ding, N. ; Goodman, S. ; Soricut, R. Conceptual Captions: A Cleaned, Hypernymed, Image Alt-Text Dataset For Automatic Image Captioning. In Proceedings of the 56th Annual Meeting of the Association for Computational Linguistics (Vol. 1: Long Papers); Gurevych, I. , Miyao, Y. , Eds.; Association for Computational Linguistics: Melbourne, Australia, 2018; pp 2556–2565.

[127] Schuhmann, C. ; Beaumont, R. ; Vencu, R. ; Gordon, C. ; Wightman, R. ; Cherti, M. ; Coombes, T. ; Katta, A. ; Mullis, C. ; Wortsman, M. ; Schramowski, P. ; Kundurthy, S. ; Crowson, K. ; Schmidt, L. ; Kaczmarczyk, R. ; Jitsev, J. LAION-5B: An Open Large-Scale Dataset for Training next Generation Image-Text Models. In Advances in Neural Information Processing Systems; Koyejo, S. , Mohamed, S. , Agarwal, A. , Belgrave, D. , Cho, K. , Oh, A. , Eds.; Curran Associates, Inc.: 2022; Vol. 35, pp 25278–25294.

[128] Ni Z., Jiao H., Fei C., Gu H., Xu S., Yu Z., Yang G., Deng Y., Jiang Q., Liu Y., Yan Y., Huang J. (2022). Evolution of Defects during the Degradation of Metal
Halide Perovskite Solar Cells under Reverse Bias and Illumination. Nat. Energy.

[129] Bertoluzzi L., Boyd C. C., Rolston N., Xu J., Prasanna R., O’Regan B. C., McGehee M. D. (2020). Mobile Ion Concentration
Measurement and Open-Access Band Diagram Simulation Platform for Halide
Perovskite Solar Cells. Joule.

[130] Lee H., Lee C. U., Yun J., Jeong C.-S., Jeong W., Son J., Park Y. S., Moon S., Lee S., Kim J. H., Moon J. (2024). A Dual Spin-Controlled
Chiral Two-/Three-Dimensional Perovskite Artificial
Leaf for Efficient Overall Photoelectrochemical Water Splitting. Nat. Commun..

[131] Ryan K., Lengyel J., Shatruk M. (2018). Crystal Structure Prediction
via Deep Learning. J. Am. Chem. Soc..

